# Necrology

**Published:** 1899-06

**Authors:** 


### NECROLOGY.

A. E. Conrow, V.M.D., M.D. At Philadelphia, in June, subsequent to operative interference for chronic appendicitis, Dr. Conrow passed away.

He was born in Chester Township, Burlington County, New Jersey. His early education was obtained in the public schools, and for several years prior to entering the Veterinary Department of the University of Pennsylvania, in 1889, he followed commercial pursuits. He graduated in 1892, and subsequently pursued a course in human medicine, obtaining a degree in medicine. He had attained a splendid practice, for a time practising both human and comparative medicine.

Always active in politics, he was elected a Democratic Freeholder in 1898 and 1899; was defeated for Senator in 1897, and was a member of the School Board.

Dr. Conrow's wife died some eight weeks prior to his demise, leaving six children, all of tender age.

Richard Kay, M.D., D.V.S. In New York City, early in April, Dr. Richard Kay died from a heart complication, the sequel of gastro-intestinal disturbance.

Born in England, his early venture in life was in the trade of a fine wood-worker, but on his arrival in America he turned his attention to cattle-trading. Entering the American Veterinary College in 1881, he graduated in the class of 1883, and spent one year as house surgeon to the American Veterinary Hospital. He subsequently pursued a course of study in human medicine and obtained the degree of M.D.

He spent several years in the employ of the Bureau of Animal Industry, but was more inclined to the field of private practice, and had spent the last ten years in this work, gaining a good practice, and was much esteemed by his clientage. While not an active Association worker, he maintained his membership in the U. S. V. M. A., and was ever proud of its progress and interested in its work. He was a member of the Masonic order. A wife and one son survive him.

Joseph R. Hodgson, Sr., D.V.S. At Brooklyn, New York, by his own hand, while mentally unbalanced, Dr. Hodgson ended his earthly career.

He was a graduate of the American Veterinary College, class of 1883, graduating in the same class as Dr. Kay. He always remained in civil practice and was not active in Association or professional circles.

ROBERT JENNINGS, JR. At Pittsburg, in June, from poisoning taken with suicidal intent, Dr. Jennings, Jr., closed his career.

A son of Dr. Robert Jennings, for many years an active practitioner in Philadelphia, and actively connected with the earlier attempts to establish a veterinary college in this city. The son spent a long term of years as an apprentice and assistant to his father, and was granted a certificate of membership in an institution styled the Pennsylvania College of Veterinary Surgeons. For many years he has maintained a large veterinary hospital in Pittsburg, and had many contracts among the draught-horse owners in the "Smoky City." Worriment from family difficulties, and the subsequent loss of prestige and recognition, preyed so heavily upon his mind as to lead him to seek relief by self-destruction.

MRS. WILLIAM DOUGHERTY. At Baltimore, Maryland, early in June, after a lingering illness, Mrs. William Dougherty, wife of Dr. William Dougherty.

Mrs. Dougherty was a Philadelphian prior to her marriage. For years her illness had been such as to despair of her life many times, but with wonderful courage and fortitude she would rally again and give rise to strong hopes of her ultimate recovery. Of a cheerful nature and sanguine temperament, she was loved by everyone who had the favor of her acquaintance, and will be much missed by a large circle of friends.

---

Dr. J. C. McKenzie, of Rochester, N. Y., has been a victim of a severe attack of inflammatory rheumatism.

Among the list of graduates of the Dental Department of the Medico-Chirurgical College of Philadelphia, class of 1899, are to be noted Veterinarian Charles M. Franz, Dr. J. B. Hagenbuch, V.M.D., of Philadelphia, graduated from the Pharmaceutical Department of the same institution.

The prize of fifty dollars for the best examination in jurisprudence at the Medico-Chirurgical College of Philadelphia was won this year by Veterinarian A. L. Lehman, a graduate of the Veterinary Department of the University of Pennsylvania, who also adds the degree of M.D. Dr. Lehman will locate at Mullen, Montana.

